# A Novel Bio-Adhesive Mesh System for Medical Implant Applications: In Vivo Assessment in a Rabbit Model

**DOI:** 10.3390/gels9050372

**Published:** 2023-05-01

**Authors:** Melinda Harman, Kevin Champaigne, William Cobb, Xinyue Lu, Varun Chawla, Liying Wei, Igor Luzinov, O. Thompson Mefford, Jiro Nagatomi

**Affiliations:** 1 301 Rhodes Engineering Research Center, Bioengineering Department, Clemson University, Clemson, SC 29634, USA; 2 School of Medicine Greenville, Prisma Health Upstate, University of South Carolina, Greenville, SC 29605, USA; 3 Circa Bioscience, Charleston, SC 29412, USA; 4 Materials Science & Engineering Department, Clemson University, Clemson, SC 29634, USA

**Keywords:** poloxamine hydrogel adhesive, “grafting to” surface modification, polymer brushes, polypropylene, surgical mesh, in vivo animal model, hernia

## Abstract

Injectable surgical sealants and adhesives, such as biologically derived fibrin gels and synthetic hydrogels, are widely used in medical products. While such products adequately adhere to blood proteins and tissue amines, they have poor adhesion with polymer biomaterials used in medical implants. To address these shortcomings, we developed a novel bio-adhesive mesh system utilizing the combined application of two patented technologies: a bifunctional poloxamine hydrogel adhesive and a surface modification technique that provides a poly-glycidyl methacrylate (PGMA) layer grafted with human serum albumin (HSA) to form a highly adhesive protein surface on polymer biomaterials. Our initial in vitro tests confirmed significantly improved adhesive strength for PGMA/HSA grafted polypropylene mesh fixed with the hydrogel adhesive compared to unmodified mesh. Toward the development of our bio-adhesive mesh system for abdominal hernia repair, we evaluated its surgical utility and in vivo performance in a rabbit model with retromuscular repair mimicking the totally extra-peritoneal surgical technique used in humans. We assessed mesh slippage/contraction using gross assessment and imaging, mesh fixation using tensile mechanical testing, and biocompatibility using histology. Compared to polypropylene mesh fixed with fibrin sealant, our bio-adhesive mesh system exhibited superior fixation without the gross bunching or distortion that was observed in the majority (80%) of the fibrin-fixed polypropylene mesh. This was evidenced by tissue integration within the bio-adhesive mesh pores after 42 days of implantation and adhesive strength sufficient to withstand the physiological forces expected in hernia repair applications. These results support the combined use of PGMA/HSA grafted polypropylene and bifunctional poloxamine hydrogel adhesive for medical implant applications.

## 1. Introduction

Synthetic and biological meshes are widely used in surgical practice, particularly for hernia repair. A hernia occurs when abdominal organs or tissues, such as the small intestine, bulge through a weakened or damaged abdominal wall. This can cause pain and discomfort, and, if untreated, can result in part of the small intestine becoming incarcerated within the abdominal wall or strangulated from inadequate blood supply [1]. Hernias are commonly located on the anterior abdominal wall (umbilical), adjacent to healed surgical scars (incisional), and in the groin region (inguinal, femoral), with groin hernias having the highest lifetime risk in both men (27–42%) and women (3–5.8%) [2]. Hernia repair surgery is the most common general surgery in the US, with over 1 million laparoscopic and open surgical procedures performed each year [3]. This high prevalence induces a significant healthcare burden of USD 3–4 billion in yearly healthcare expenditures related to hernia treatment [4,5].

More than 90% of incisional and inguinal hernias involve placing surgical mesh fabricated from medical-grade polymers at the site of the hernia [6]. Polypropylene (PP) is the most common synthetic mesh material and is used in two out of every three inguinal hernia repairs [7]. During abdominal hernia repair, polymer surgical meshes are often fixed to abdominal tissues by mechanical devices such as sutures, staples, or tacks. However, these traumatic devices can cause substantial post-operative pain and are difficult to employ laparoscopically. As such, the fixation of surgical meshes with tissue adhesives has been pursued as an atraumatic fixation method, and there is evidence for a reduction in lingering pain after surgery [8].

Fibrin sealant is a biodegradable and biocompatible combination of human fibrinogen and thrombin that polymerizes into a fibrin matrix [9]. The use of fibrin sealants such as the commercially available Tisseel^®^ or Tissucol^®^ (Baxter Healthcare, Deerfield, IL, USA) has been shown to potentially reduce pain and improve overall post-operative outcomes compared to standard suture or tack fixation for inguinal hernia repair [10,11,12]. However, fibrin sealants do not provide immediate fixation of the polymer mesh [13], which may result in improper tissue ingrowth into the mesh. This limitation leads to an increased possibility of hernia recurrence due to curling of the mesh edges and mesh slippage, which necessitates concomitant insertion of mechanical devices and patient exposure to the risks of lingering pain [14,15,16,17,18]. Implanted polymer meshes also can trigger an inflammatory foreign body reaction that persists long after surgery and is a significant additional source of long-term pain [19]. Strategies to attenuate the inflammatory response to implanted mesh have been investigated, including coating the implants with isolated extracellular matrix proteins [20,21] or hydrogels [22,23,24], but this is not yet common practice for hernia repair. There is, therefore, a critical need for technology that ensures rapid fixation for implantable mesh with a reduced inflammatory response and an adhesive that has the appropriate balance of strength and biodegradability for proper healing and long-term repair.

**Figure 1:**
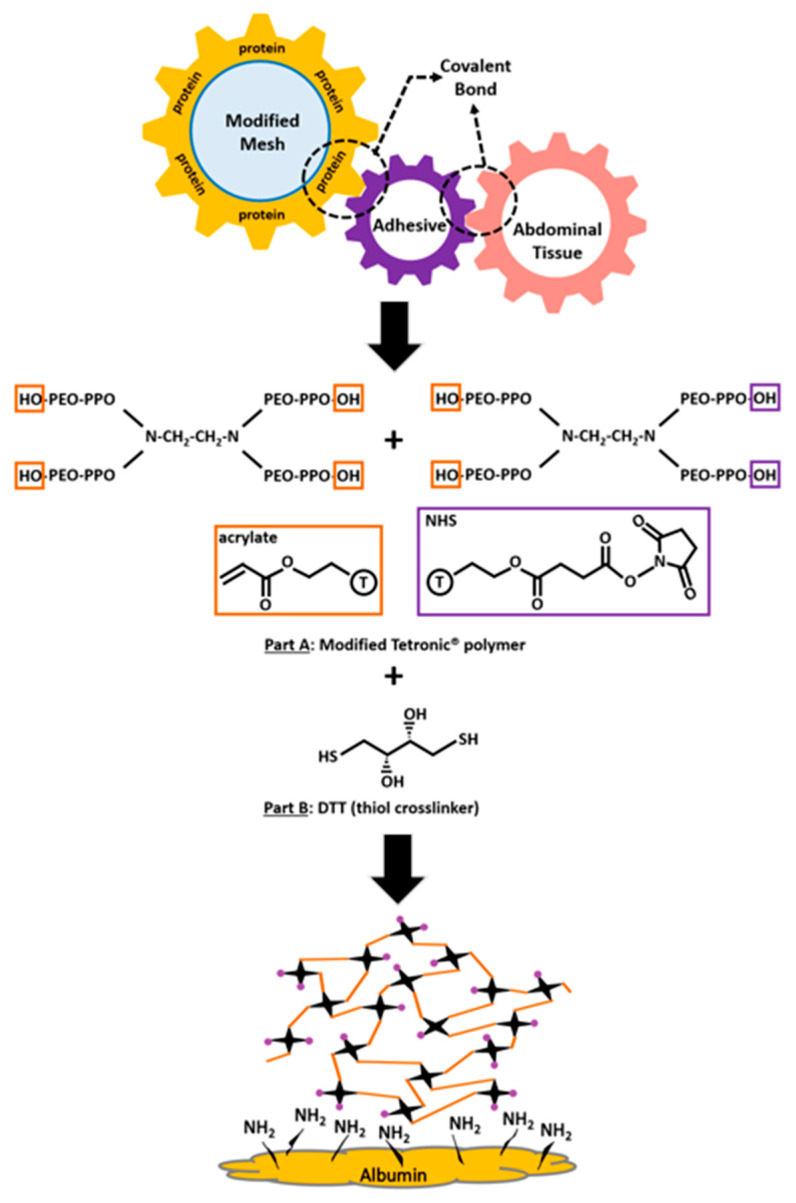
The modified bi-functional Tetronic^®^ 1107 (T1107) adhesive interacts with the modified polymer mesh and abdominal tissues. The four-arm structure of Tetronic^®^ polymer is bi-functionalized with acrylates for chemical crosslinking of the hydrogel for bulk strength and N-hydroxysuccinimide (NHS) for reaction-free thiols present in the bio-plastic albumin protein layer on the mesh surface and extracellular matrix proteins in tissues.

Polymeric (synthetic and extracellular matrix-derived) hydrogels are widely used in commercially available medical products, including drug delivery systems, wound dressings, and tissue engineering scaffolds due to tunable properties such as biodegradation, flexibility, and fast gelation [25,26,27]. We have previously developed and characterized hydrogel adhesives that consist of a modified Tetronic^®^ T1107 (BASF Corporation, Florham Park, NJ, USA) four-arm poly (propylene oxide)-poly (ethylene oxide) (PPO-PEO) block polymer for swelling, degradation, gelation temperature, mechanical properties, and biocompatibility [28,29,30,31,32]. T1107 solution (30% w/v) exhibits gelation at temperatures of 20 °C–22 °C [31]. When Tetronics having different molecular masses (T1107 v. T904 v. T304) are mixed at specific ratios, these polymer mixtures exhibit reverse thermal gelation at physiological temperatures such that the solution remains at liquid at room temperature and forms a gel when applied to body-temperature tissues [28,31]. This heat sensitivity (thermal gelation property) is advantageous because it prevents the gel from flowing away from the site of surgical application. These polymer mixtures can be combined with Michael-type crosslinking reaction (covalent crosslinking via a thiol crosslinker) to achieve a “tandem gelation” process [33] and increase the peak storage modulus from 36.25 ± 4.88 kPa to 52.61 ± 11.26 kPa [31]. After this chemical reaction and exposure to body temperatures, the hydrogel remains crosslinked and will not return to a solution phase at lower temperatures. Taking advantage of the 4-arm structure of Tetronic^®^, bi-functionalization of the polymer has been achieved with acrylates for chemical crosslinking of the hydrogel for bulk strength and N-hydroxysuccinimide (NHS) for reaction with tissue amines to further improve the adhesive bond strength with tissues (Figure 1) [29]. This hydrogel adhesive has been shown to be non-toxic to cells in culture, have relatively low swelling (2–3%), and is degradable (at the ester bonds of the crosslinked molecules) under aqueous conditions over a 2-week period (slow in the beginning and sudden at later time points) [29,31]. The material was shown to be safe in vivo when tested as a tissue adhesive for the treatment of small soft-tissue wounds in rats, eliciting a controlled inflammatory response in vivo without provoking unwanted tissue adhesions when used on the large intestine [29,30]. It was also previously shown to exhibit adhesive strength that exceeds 70 kPa via mechanical interdigitation and covalent bond formation with tissue amines [30,32].

To evaluate the applicability of this hydrogel adhesive to hernia repair surgery, it was tested with multiple types of PP mesh, but the adhesive strength was lower than the adhesive strength for collagen tissues [29,32]. It was speculated that the adhesive strength was limited by the hydrophobicity of PP monofilaments and the lack of covalent bond formation [32]. Thus, we hypothesized that surface modifications of PP mesh with the introduction of serum proteins might improve the adhesive strength by achieving covalent bonds. We investigated a surface modification technique that involves grafting permanent covalent functional groups onto materials to form a protein coating [34,35]. Poly-glycidyl methacrylate (PGMA), which contains an epoxy group in each repeating unit, can be used as an anchoring layer for grafting of macromolecules (including proteins) on the surface of organic and inorganic materials, including medical devices [34,35,36,37,38,39,40,41]. The PP surfaces of mesh are activated with plasma to provide radicals, and these radicals react with oxygen and water, forming functional groups for depositing the PGMA layer. The PGMA layer epoxy groups then react with human serum albumin (HSA) to form a robust three-dimensional bio-plastic protein layer of albumin on the PP surface that is resistant to bacterial adhesion in cell culture and stable for up to four months in vivo [42]. Lap shear testing demonstrated that this PGMA/HSA surface modification significantly improved the adhesive strength for meshes attached to our poloxamine hydrogel tissue adhesive [32].

In this study, we compared the in vivo performance of our novel bio-adhesive mesh system, consisting of PGMA/HSA grafted polymer mesh secured with our bio-adhesive hydrogel, versus the performance of unmodified PP mesh secured with Tisseel^®^ fibrin sealant (hereafter, fibrin-fixed PP mesh) in a rabbit abdominal hernia repair model. A retromuscular repair was performed on five rabbits that mimicked the totally extra-peritoneal (TEP) surgical technique used in humans for hernia repair. After 42 days in situ, we characterized mesh slippage and contraction using gross assessment and imaging. Following the explant on day 42, we measured mesh fixation strength using tensile mechanical testing and assessed biocompatibility using histology.

## 2. Results and Discussion

### 2.1. Preparation and Handling of the Bio-Adhesive Mesh System

**Figure 2:**
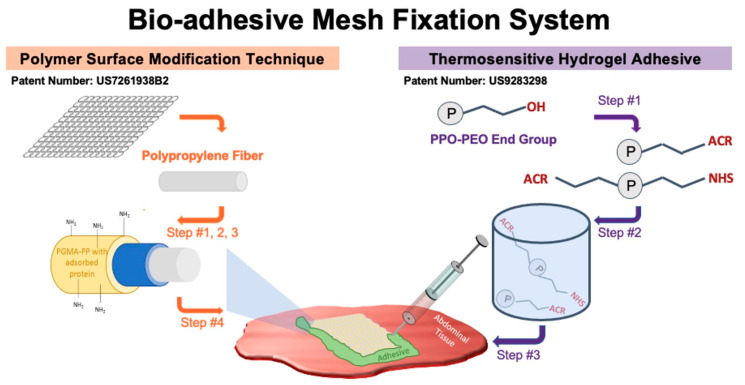
The bio-adhesive mesh system makes use of two patented technologies—a polymer surface modification technique and a thermo-responsive hydrogel adhesive. The polymer surface modification technique created a bio-plastic layer on the PP mesh and involved steps for: (1) plasma treatment, (2) activating the PP surface with functional groups, (3) dip coating in HSA, and (4) annealing and drying before use. The hydrogel adhesive was generated using: (1) the acrylation (ACR) process to chemically crosslink the polymer within the hydrogel; (2) the NHS process to facilitate binding to tissue amines; and (3) chemical crosslinking using DTT as the thiol donor crosslinker.

Surface modifications are part of an emerging trend toward improved bio-functionality of surgical mesh polymers [43,44] and biologic coatings such as gelatin, chitosan, purified collagen, and extracellular matrix are commonly pursued to improve mesh performance [21,23,45,46,47,48]. Similarly, current research on tissue adhesives [49] focuses on improving mechanical properties for internal applications, including the exploration of multi-functional hyperbranched polymers and various biomimetic molecules to support standalone adhesion without the need for supplemental sutures. One of our objectives for the design of the novel bio-adhesive mesh system was to evaluate the combined use of PGMA/HSA grafted PP surgical mesh and a thermo-responsive hydrogel adhesive for medical implant applications (Figure 2). Both technologies have proven useful in other in vivo applications, but their combined in vivo use for rapid fixation of an implantable mesh has not been reported to date.

We successfully anchored a bio-plastic HSA protein layer onto the PP mesh surface using a “grafting to” surface modification technique [34,35,36,37,41]. Briefly, the PGMA/HSA surface modification process involved air plasma treating and activating the PP mesh with reactive functional groups, dip coating and annealing PGMA to provide a uniform and homogeneous macromolecular anchoring layer, and dip coating and annealing the HSA to form a three-dimensional bio-plastic layer on the surface of PP mesh. This process produced a submicron protein layer that did not block the mesh pores, thereby maintaining the macro-porosity necessary for tissue integration during healing [50,51].

We successfully prepared the bifunctional poloxamine hydrogel adhesive following established methods [30,31]. Briefly, Tetronic^®^ 1107 (T1107), a bifunctional poloxamine four-arm poly (propylene oxide)-poly (ethylene oxide) (PPO-PEO) block co-polymer was first partially acrylated to form T1107-ACR and unreacted arms were further modified by coupling NHS to form T1107-ACR-NHS [28,29,30]. These polymer (T1107-ACR and T1107-ACR-NHS) solutions (30% w/v%) were combined in a 75 wt%:25 wt% ratio and then chemically crosslinked with a thiol donor crosslinker solution (DTT) mixed at a thiol to acrylate molar ratio of 1:1 at the point of use. This process produced a hydrogel adhesive with thermal gelation properties that support precise surgical application and strongly bind tissue surfaces and the protein-grafted polymer mesh [29,30,32].

In the current study, the bio-adhesive mesh system was able to withstand necessary preparations for surgical use and handling, and gelation was noted during the implantation surgery. That is, the PGMA/HSA grafted meshes withstood ethylene oxide sterilization methods common for surgical implant materials, they were not sticky, and their cutting and handling characteristics were not different from unmodified meshes. We successfully controlled the thermo-responsive behavior of the hydrogel adhesive at the point of use by maintaining the hydrogel in its solution phase on ice (~4 °C), enabling efficient delivery using a standard pipette for precise application to the mesh surface by the surgeon. There was rapid gelation of the hydrogel adhesive observed at the time of surgery, with chemical crosslinking presumably occurring after the hydrogel was applied to the tissues (e.g., we observed the bio-adhesive mesh for approximately 15 min after implantation when one surgery was delayed while a new fibrin sealant applicator was prepared). These results strengthened our previous bench tests of PGMA/HSA PP mesh, confirming the strong chemical bonds between the PGMA epoxy groups and the plasma-activated PP surface and between amino and carboxyl groups in HSA and remaining epoxy groups in PGMA [32]. When considered together with our previous studies [28,29,30,31,32], these data provide strong evidence supporting in vivo use of the bio-adhesive mesh system.

### 2.2. In Vivo Animal Model

**Figure 3:**
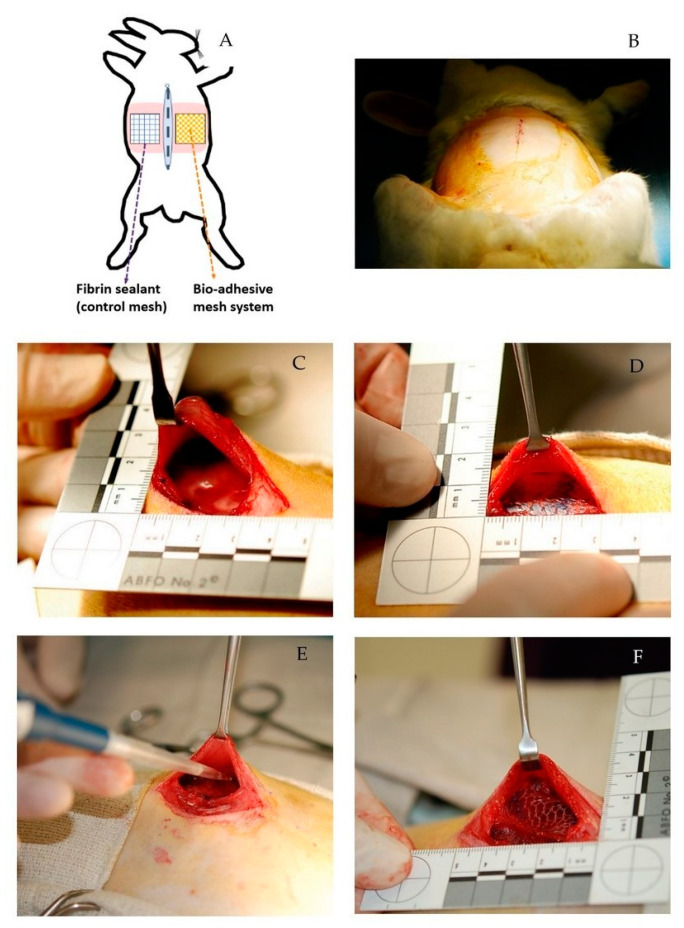
(**A**,**B**) We surgically created a 5 cm incision along the linea alba (dashed line) and intermuscular planes (pink regions) on the left and right abdominal wall. (**C**,**D**) We implanted the mesh-fixation treatments within the intermuscular planes, with fibrin-fixed PP mesh on the left side and bio-adhesive mesh on the right side. All mesh were similarly oriented with the blue fibers perpendicular to the linea alba. (**E**,**F**) The surgeon efficiently delivered the hydrogel adhesive using a standard pipette for precise application to the mesh surface, and rapid gelation of the hydrogel adhesive was observed at the time of surgery.

Using a rabbit animal model, which is one of the most common for abdominal wall hernia research [52], we replicated a retromuscular repair to mimic the totally extra-peritoneal (TEP) surgical technique commonly used in humans for abdominal hernia repair [53]. Because our primary interest was to assess the fixation and biocompatibility of the bio-adhesive mesh system, this model avoids the transection of the peritoneum and the confounding tissue adhesions that develop with exposure of the peritoneal cavity to PP [14]. A surgeon experienced in human and animal surgery used that surgical approach to create intermuscular planes between the transversus abdominis and internal oblique muscles lateral to each side of the linea alba and implantation of one coupon of the bio-adhesive mesh and one coupon of the unmodified PP mesh fixed with fibrin sealant (Figure 3). There were no wound-healing complications, and no animals were lost prior to the planned study endpoint (42 days).

The rabbits were euthanized on day 42, and the skin was resected to expose the implant sites. The implant sites were assessed and photographed in situ and immediately harvested en bloc with the surrounding muscular tissue margins. The onset of muscle stiffening due to rigor mortis was not physically evident in any rabbit. Loose adhesions between the abdominal wall and organs were evident at explantation in one rabbit (R2); there were no other notable adhesions in the other rabbits. We did not observe any remnant of the hydrogel at the time of sample retrieval or while we carefully cut each mesh–tissue block to yield four 1 × 3 cm sized sections for mechanical testing (n = 2 muscle tissue with mesh and n = 1 muscle tissue margin without mesh) and histological analysis of biocompatibility (n = 1). We maintained tissue sections either moist on ice for subsequent mechanical testing that we completed within approximately 2 h of resection or in formalin for subsequent histological processing.

### 2.3. Mesh Slippage and Contraction

**Figure 4:**
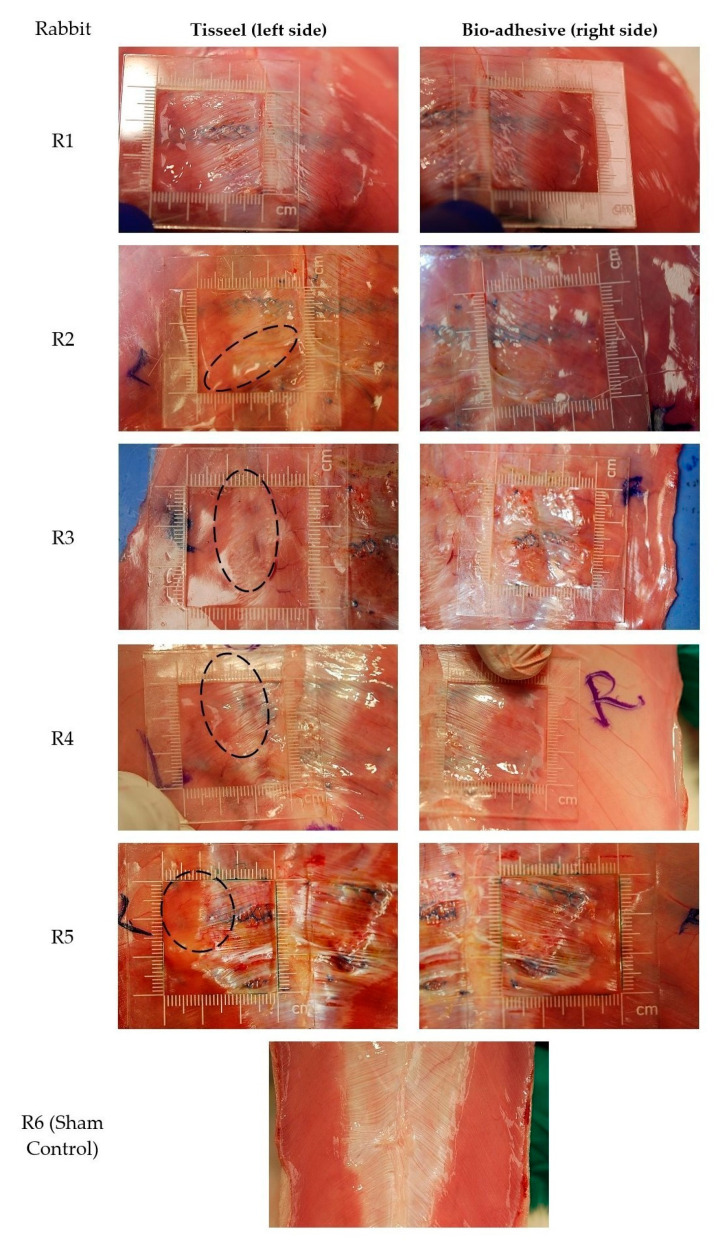
Calibrated digital images of mesh in situ with the transparent grid overlay denoting nine 1 × 1 cm grid zones. Gross bunching of the fibrin-fixed PP mesh was evident in four rabbits (R2–R5), with bunching generally occurring on the edge furthest from the linea alba and occupying two or more zones in each animal (denoted with dashed circles). None of the bio-adhesive mesh had gross bunching.

We grossly assessed mesh slippage and contraction in situ at the time of euthanasia and in calibrated digital images (Figure 4). We defined mesh slippage and contraction as gross bunching in the mesh causing distortion of the usual flat profile of the mesh. Rigor mortis did not confound these assessments since muscle tissues resected from New Zealand white rabbits experienced only small variations in the stress–strain response when tested within 7 h of euthanasia, prior to the onset of rigor [54]. Visual evidence of bunching was assessed in each of nine 1 × 1 cm grid zones denoted by the transparent grid overlay. There was no bunching for 5 of 5 (100%) of the bio-adhesive mesh and for 1 of 5 (20%) of the fibrin-fixed PP mesh. In contrast, 4 of 5 (80%) of the fibrin-fixed PP mesh had notable evidence of bunching resulting in distortion in at least 2 grid zones, corresponding to >22% of the mesh surface area. These observations confirm the benefit of rapid gelation of the hydrogel adhesive at physiological temperatures, providing for tissue attachment between the intramuscular planes and tissue integration within the mesh pores, as noted histologically in our subsequent analysis of biocompatibility. The observed bunching and distortion of the fibrin-fixed PP mesh is consistent with a lack of initial fixation, as reported by Jenkins et al. using a variety of polymer mesh implanted in a rabbit model [13]. During hernia repair applications, the quick gelation and immediate fixation of this bio-adhesive mesh system may contribute to stable mesh fixation compared to the fibrin sealant.

### 2.4. Mesh Fixation

**Figure 5:**
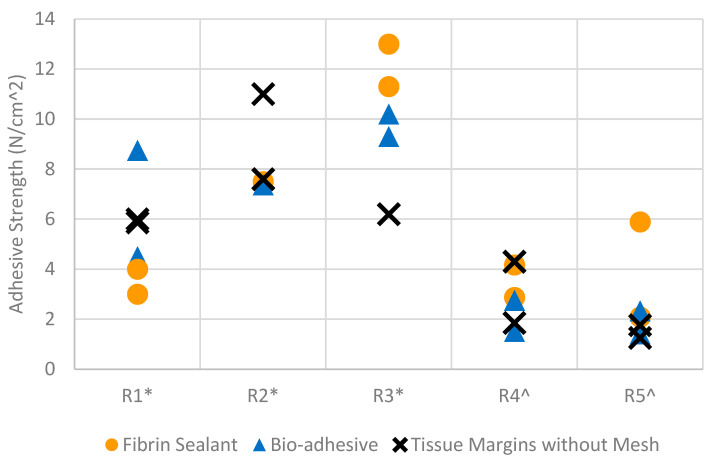
Adhesive strength for lap shear tests (*) and peel tests (^) for the five rabbits (R1–R5) implanted with fibrin-fixed PP mesh and bio-adhesive mesh. Due to small sample dimensions, only one type of test was possible for mesh specimens from each rabbit.

We measured the adhesive strength of the bio-adhesive mesh and the fibrin-fixed PP mesh using lap shear and T-peel mechanical testing. We defined adhesive strength as the peak load divided by the gauge length at the tissue/mesh/adhesive interface [32]. Both methods of fixation allowed for tissue ingrowth into the mesh pores, resulting in mechanical interlock at the mesh–tissue interface and comparable mesh fixation after 42 days of implantation (Table 1, Figure 5). Adhesive strength using the bio-adhesive mesh system after 42 days of implantation was equivalent to the control mesh with fibrin fixation (paired t-test, *p* > 0.05). Moreover, adhesive strength for the tissues with mesh was not significantly different from the tissue margins without mesh (ANOVA *p* > 0.05). Adequate adhesive strength for PP mesh fixation is critical for successful hernia repair, requiring both rapid and strong initial fixation to maintain the mesh in the intended position and withstand physiological forces (2–3 N) [32]. In the current study, we can infer that the bio-adhesive mesh system possesses sufficient adhesive strength for hernia repair applications.

### 2.5. Mesh Biocompatibility

**Figure 6:**
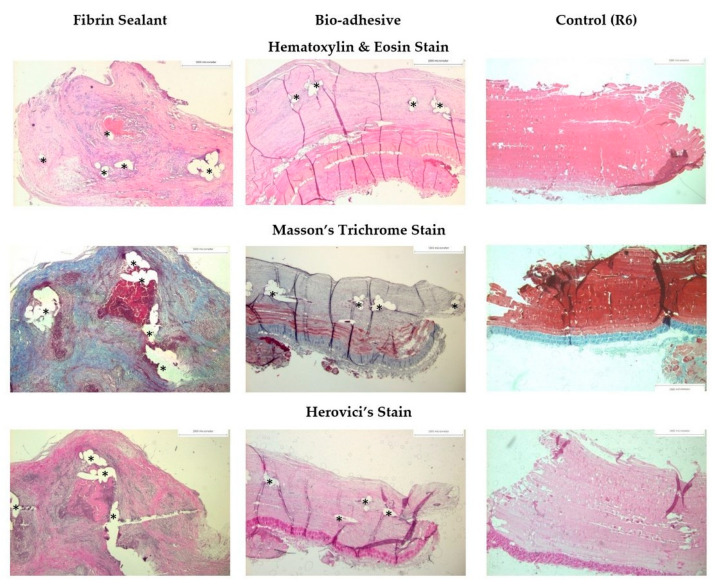
Representative histology for the fibrin-fixed PP mesh and the bio-adhesive mesh system, with all sections oriented with the peritoneal tissues at the top of each image and the subdermal muscle tissues at the bottom of each image. Scale bar = 1000 microns. * = mesh fibers.

**Figure 7:**
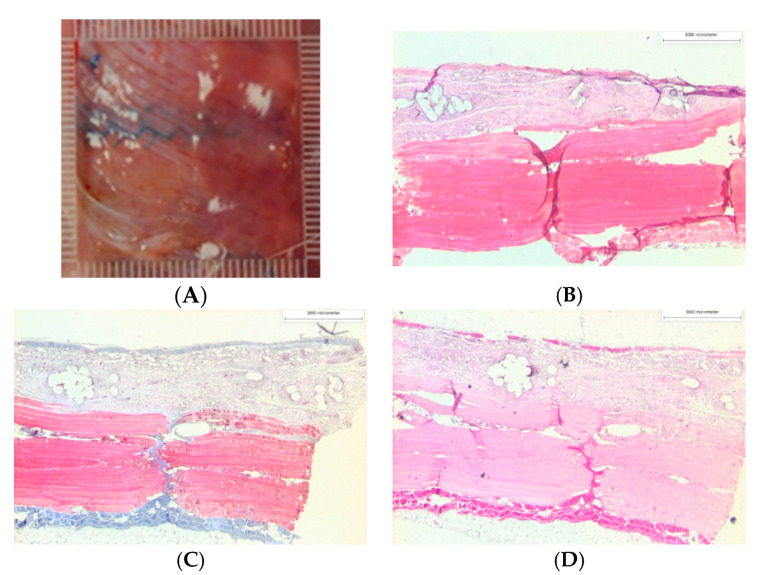
(**A**) Representative gross photo of a flat smooth bio-adhesive mesh system after 42 days implantation and histology images for (**B**) hematoxylin and eosin, (**C**) Masson’s trichrome, and (**D**) Herovici stains.

The implant sites were harvested en bloc and 1 × 3 cm sized sections were processed, sectioned, stained, and imaged for histological analysis. Stains included hematoxylin and eosin to identify signs of an inflammatory response and foreign body reaction, Masson’s trichrome to identify fibrous connective tissues and degree of collagen synthesis, and Herovici’s stain to differentiate immature and mature collagen (Figure 6 and Figure 7). The bio-adhesive mesh system did not have different biocompatibility results compared to fibrin-fixed PP mesh after 42 days of implantation. The average percentage area of nuclei, collagen, and muscle in tissue sections from bio-adhesive mesh fixation was not significantly different than those with fibrin-fixed PP mesh (Table 2) after normalization to R6 (*p* > 0.05, *t*-test). Macrophages and foreign body giant cells infiltrated in the tissue surrounding the mesh implants likely reflect the known inflammatory response to PP materials, and it is consistent with other animal models with PP mesh implants [53]. Given the relatively short degradation times for both adhesives, within 10–14 days for Tisseel^®^ fibrin sealant [9] and over a 2-week period for the bio-adhesive [29,31], tissue reactions specific to the fibrin sealant and bio-adhesive were not expected in this animal model.

There was evidence of tissue ingrowth within the inter-fiber mesh pores for both the fibrin-fixed PP mesh and bio-adhesive mesh (Figure 6), consistent with tissue integration into the large pore mesh. Overall, collagen deposition was similar for both the fibrin-fixed PP mesh and bio-adhesive mesh and the unimplanted control tissue, with thick bands of bold blue mature collagen noted along the periphery defined by the surgical intermuscular placement of the mesh. A 2-layer muscle pattern was highly evident as having both longitudinal and cross-sectional structures. Collagen was lightly stained and appeared loosely packed in sections of the bio-adhesive mesh compared to the fibrin-fixed PP mesh (Figure 6 and Figure 7). Qualitatively, tissues from rabbits with bunching of the fibrin-fixed PP mesh displayed greater collagen organization even though the area% was similar to tissues without bunching (Figure 6). Although there was evidence of fibrous connective tissues and collagen dispersed within the inter-fiber mesh pores (Figure 6 and Figure 7), we did not observe notable “bridging” across mesh fibers for either the fibrin-fixed PP mesh or bio-adhesive mesh in the current study. “Bridging” occurs when smaller pores become filled with fibrotic tissues and inflammatory cells and is generally undesirable [50,51].

## 3. Conclusions

In the current study, we demonstrated a novel bio-adhesive mesh system utilizing a PGMA/HSA grafted PP surgical mesh and a thermo-responsive hydrogel adhesive. We evaluated the surgical utility and in vivo performance of the bio-adhesive mesh system in a rabbit model with retromuscular repair mimicking the totally extra-peritoneal surgical technique commonly used in humans for abdominal hernia repair. The bio-adhesive mesh system was able to withstand necessary preparations for surgical use and handling, and sterilization methods were sufficient to avoid infection. The thermo-responsive behavior of the hydrogel adhesive was demonstrated during surgery by maintaining the solution on ice prior to efficient delivery via pipette for surgical application onto the warm tissues and rapid gelation. Compared to unmodified PP mesh fixed with fibrin sealant, our bio-adhesive mesh system exhibited superior fixation without gross bunching or distortion that was observed in the majority (80%) of the fibrin-fixed PP mesh. This was evidenced by tissue integration within the mesh pores of the bio-adhesive mesh after 42 days implantation, and adhesive strength were sufficient for withstanding physiological forces expected in hernia repair applications. These results support the combined use of PGMA/HSA grafted PP and bifunctional poloxamine hydrogel adhesive for medical implant applications. Ongoing work includes using the bio-adhesive mesh system for the fixation of other polymer mesh materials and completing an additional animal study comparing mesh fixation with commercially available cyanoacrylate tissue glues.

## 4. Materials and Methods

### 4.1. Materials

#### 4.1.1. Polymer Mesh

Commercially available Prolene^®^ Soft polypropylene (PP) surgical mesh sheets were purchased from Ethicon (Somerville, NJ, USA). These surgical mesh sheets are warp-knitted PP fibers and have well-characterized pore structures (macro porosity >60% and pore diameter >1 mm) [54]. This is a nonabsorbable synthetic surgical mesh cleared by US FDA as a hernia device.

#### 4.1.2. Polymer Surface Modification

Glycidyl methacrylate, azobisisobutyronitrile (AIBN), human serum albumin (HSA), and potassium phosphate buffer were purchased from Sigma-Aldrich (St. Louis, MO, USA). Methyl ethyl ketone (MEK), chloroform, and diethyl ether were purchased from VWR (Suwanee, GA, USA).

#### 4.1.3. Hydrogel Adhesive

Tetronic^®^ 1107 (T1107, molecular mass 15 k Da, HLB:18–23) was donated by BASF Corporation (Florham Park, NJ, USA). Acryloyl chloride, Celite fine 500, 4-dimethylaminopyridine (DMAP), succinic anhydride, N-hydroxysuccinimide (NHS), 4-methoxyphenol (99%), calcium hydride, tetrahydrofuran (THS) and chloroform were purchased from Sigma-Aldrich (St. Louis, MO, USA). Toluene (HPLC grade), diethyl ether (anhydrous, BHT stabilized), and anhydrous sodium sulfate were purchased from Fisher Scientific (Fair Lawn, NJ, USA). Dichloromethane (DCM) (HPLC grade), triethylamine (TEA), ditiothreitol (DTT), sodium bicarbonate, calcium hydride and d-chloroform (CDCl_3_) were purchased from Acros Organics (Pittsburg, PA, USA) and 1-(3-dimethylaminopropyl)-3-ethylcarbodiimide hydrochloride (EDC) from Tokyo Chemical Industry (Tokyo, Japan). Unless otherwise specified, all chemicals were of analytical grade and were used as received. Double-deionized ultra-filtered water was used throughout this study.

#### 4.1.4. Fibrin Sealant

Commercially available Tisseel^®^ fibrin sealant was purchased from Baxter Healthcare (Round Lake, IL, USA).

#### 4.1.5. Animal Model

Six male rabbits (Oryctolagus cuniculus species, New Zealand White strain), each weighing 2–3 kg, were purchased from approved vendors through the Clemson University Godley Snell Research Center animal facility. PDS*II (polydioxanone absorbable, 3-0) suture was purchased from Ethicon (Somerville, NJ, USA).

#### 4.1.6. Biocompatibility

Glass slides (Colorfrost Plus) were purchased from Richard-Allan Scientific (Kalamazoo, MI, USA). Hematoxylin and eosin stains were purchased from Newcomer Supply (Middleton, WI, USA). Masson’s trichrome was purchased from Sigma-Aldrich (St. Louis, MO, USA). Herovici collagen stain was purchased from American MasterTech Scientific (St. Lodi, CA, USA).

### 4.2. Methods

#### 4.2.1. Polymer Mesh

Using an aseptic technique, the PP mesh sheets were cut into 3 × 8 cm strips in preparation for the polymer surface modification. Following PGMA/HSA grafting, the strips were further reduced to a 3 × 3 cm coupon in preparation for implantation. Similarly, unmodified PP mesh sheets (controls) were cut into 3 × 3 cm coupons using aseptic techniques. Subsequently, all mesh intended for implantation (n = 5 coupons of PGMA/HSA grafted mesh and n = 5 coupons of unmodified mesh) were sterilized in ethylene oxide gas and sealed in Tyvek pouches until used in the animal experiment.

#### 4.2.2. Preparation of PGMA/HSA Grafted Polypropylene Mesh

The PGMA/HSA surface modification involved the fabrication of an albumin protein layer on the PP mesh fibers (Figure 2). That is, it used a “grafting to” method, with PGMA providing a uniform and homogeneous macromolecular anchoring layer (polymer brushes) for grafting the HSA protein to the PP mesh surface [34,35]. Briefly, the process for PGMA/HSA grafting creates a bio-plastic PP mesh and involves steps for (1) plasma treatment, (2) activating the PP surface with functional groups, (3) dip coating in a 3% (w/v) solution of HSA, and (4) annealing and drying before use.

The PGMA and HSA solutions were prepared according to our previously published methods [39,41,42]. Briefly, poly(glycidyl methacrylate) (PGMA) was prepared via free radical polymerization of glycidyl methacrylate in MEK at 45 °C. AIBN was used as an initiator, and the polymer obtained was purified by multiple precipitations from MEK solution in diethyl ether. A solution of 0.5% (w/v) PGMA powder (Mn = 176,000 g/mol) in chloroform was placed on an orbital shaker until the PGMA was fully dissolved and then filtered using a 0.45 µm PTFE filter. A solution of 3% (w/v) HSA in phosphate buffer was prepared.

The PP mesh strips were plasma treated for 10 min at 700 V DC, 15 mA DC, 10.5 W (Plasma Cleaner/Sterilizer, Harrick, Pleasantville, NY, USA) immediately followed by soaking in deionized water for 30 min to activate the PP surface with hydroxyl, carboxylic acid, and nitric oxide functional groups. After 30 min, the mesh strips were oven dried (80 °C) for 10 min and purged under ultrahigh purity nitrogen until fully dried. The plasma-treated mesh strips were dip coated (Meyer Fientechnik, Gottingen, Germany; D-3400) in the 0.5% (w/v) PGMA solution at a speed of 300 mm/min for a 10 min immersion time and then annealed at 120 °C for 10 min to enhance the bonding between the PGMA and PP mesh. After annealing, the PGMA-modified meshes were immersed in the 3% (w/v) HSA for 2 h. The PGMA/HSA grafted mesh strips were dried at room temperature for 12 h, followed by annealing for 2 h at 120 °C. 

#### 4.2.3. Infrared Spectroscopy of PGMA/HSA Bonding Strength

The bonding strength between the PGMA and HSA on the PGMA/HSA grafted mesh surface was confirmed by ATR-FTIR analysis (Nicolet Magna 550 FTIR spectrometer equipped with a SpectraTech Endurance Foundation Series Diamond ATR, Thermo, Waltham, MA, USA). Prior to FTIR, the PGMA/HSA grafted mesh samples were washed by immersing in phosphate buffer at 37 °C and pH 7.4 on an orbital shaker for 24 h to remove any HSA that was not covalently bonded to the PGMA followed by immersing in chloroform at room temperature to remove any PGMA that was not covalently bonded to the PP surface. Unmodified PP mesh was used as a control.

**Figure 8:**
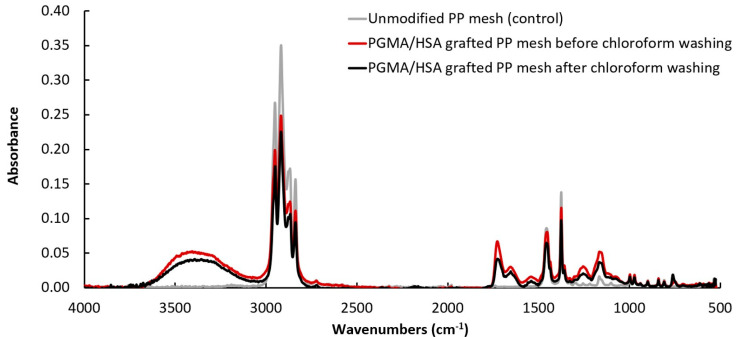
FTIR spectra of PGMA/HSA grafted PP mesh before and after washing by chloroform and unmodified PP mesh (control).

We have previously shown that the PGMA/HSA surface modification yields chemical bonds formed between the amino and carboxyl groups in albumin and the epoxy group in PGMA [32,55]. The presence of PGMA was evident by absorbance around 1730 cm^−1^ (stretching of C O groups) (Figure 8). The conversion of epoxy groups in PGMA was evident by the decrease of absorbance around 910 cm^−1^. The presence of HSA was evident by absorbance at 1541 cm^−1^ (amide II C H stretching and N H bending), around 1653 cm^−1^ (bending of N H groups), and absorbance from 3300 to 3500 cm^−1^ (stretching of amide A N H groups), which is not observed in unmodified PP controls (Figure 8). There were no differences in wave number peaks comparing before and after washing by chloroform.

#### 4.2.4. Gel Permeation Chromatography (GPC) of the PGMA (Figure 9)

The molecular mass of the PGMA was determined using gel permeation chromatography with a Styragel HR 5E column (Breeze System, Waters Corp., Milford, MA, USA). We calibrated the GPC using polystyrene standards. We dissolved the PGMA in chloroform and filtered it before GPC analysis. Sample injection was performed with an autosampler using an injection volume of 50 microliters per sample. The flow rate through the column was set to 1 mL/min with the column temperature held constant at 33 °C. The effective molecular mass range of the column was 2000 Daltons to 4 × 10^6^ Daltons. The resulting PGMA mass-average molecular mass (Mw) was 892,484 g/mol, and the polydispersity index (PDI) was 4.3. We associated the relatively high PDI with the presence of lower molecular mass fractions in the PGMA, which is represented by the number-average molecular mass (Mn) of 208,824 g/mol from GPC results.

#### 4.2.5. Nuclear Magnetic Resonance (NMR) Spectroscopy of the PGMA

**Figure 10:**
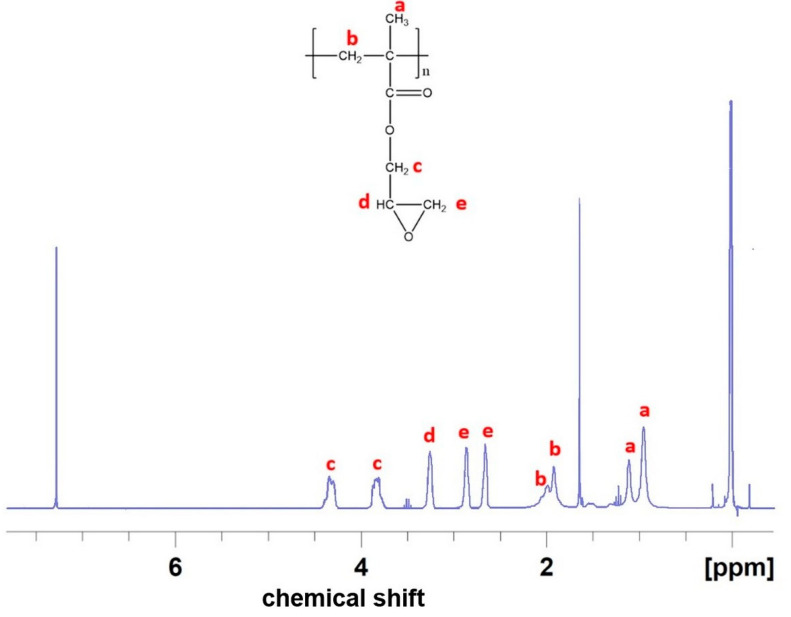
Structure and NMR spectrum of PGMA with protons indicated by the labeled letters **a** to **e**.

The ^1^H NMR (300 MHz) of the PGMA was recorded on a Bruker Advance II Spectrometer. The sample was dissolved in deuterated chloroform (d-chloroform) before the analysis. Overall, we observed peaks at 0.8–1.2 ppm (a) [3H, -CH_3_], 1.8–2.1 ppm (b) [2H, -CH_2_], 3–2.5 ppm (e) and 3.5–4.7 ppm (c) [4H, -CH_2_], and 3.3 ppm (d) [1H, -CH]. It confirmed the synthesis of the targeted PGMA (Figure 10).

#### 4.2.6. Overview of Hydrogel Adhesive Preparation

Two polymers used for the bifunctional poloxamine hydrogel adhesive were synthesized from T1107 following our previously published methods [28,29,30,31]. Briefly, for the first polymer, the hydroxyl end on four arms of T1107 were reacted with acryloyl chloride to form T1107-ACR. For the second polymer, the NHS groups were added to partially (50%) acrylated T1107-ACR to form T1107-ACR-NHS. The ACR group was used to chemically crosslink the polymer within the hydrogel, and the NHS group facilitated binding to tissue amines [30].

#### 4.2.7. Proton NMR Spectroscopy of the Hydrogel Adhesive

**Figure 11:**
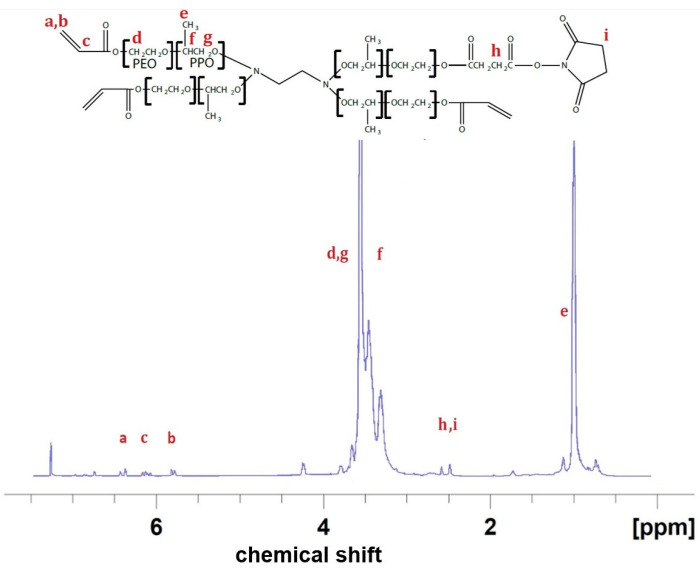
Structure and NMR spectrum of T1107-ACR NHS with protons indicated by the labeled letters a to i.

The composition and conversion efficiency of T1107-ACR and T1107-ACR NHS was assessed by Proton NMR in d-chloroform. The spectrum consisted of 4 sections, methyl (PPO, ~1 ppm), methylene (PEO, ~3.5 ppm), functional ends (NHS, Succinic acid, ~2.7 ppm), and acryloyl (C=C, ~6 ppm). T1107 has approximately 19 propylene oxide monomer units and 60 ethylene oxide monomer units for each arm and the NMR peak areas were 298 and 57, respectively, consistent with predicted calculated values (Figure 11). Qualitatively, the functional groups were all shown in the spectrum (succinic, NHS, and acryloyl) with acceptably high acrylation rates and NHS rates. Quantitatively, the modification rates for these three moieties were 19%, 29.3%, and 99%, respectively.

#### 4.2.8. Preparation of Hydrogels and Adhesives

The bifunctional poloxamine hydrogel adhesive was made using polymer solutions consisting of T1107-ACR and T1107-ACR-NHS in 1x PBS, with the final formulation containing a 30 wt% mixture of 75 wt% T1107-ACR (ACR conversion: 92%) and 25 wt% T1107-ACR-NHS (ACR conversion: 30%). The polymer solution was left in a cold room under continuous orbital mixing overnight and then stored refrigerated and in vials covered in aluminum foil to avoid light exposure. The polymer solution required chemical crosslinking via a Michael-Type addition reaction using DTT as the thiol donor crosslinker. DTT solutions were prepared fresh at a concentration of 3 mg of DTT in 10 μL of PBS. The polymer solution and DTT solutions were transported to the animal facility on ice in an insulated cooler.

To make the bifunctional poloxamine hydrogel adhesive, polymer solutions and DTT solutions were mixed at a thiol to acrylate molar ratio of 1:1, and vortexed for at least 5 s immediately before surgical use in the implantation of the mesh. Because the thermal gelation temperature of the poloxamine hydrogel adhesive was room temperature, the bifunctional poloxamine hydrogel adhesive was kept on ice (~4 °C) at the point of use [29,31].

#### 4.2.9. In Vivo Animal Model

All animal work was reviewed and approved by the Institutional Animal Use and Care Committee (IACUC) at Clemson University (protocol number AUP2019-022). The experimental work was conducted in compliance with ethical principles and animal care standards established by the US Animal Welfare Act regulations and US Public Health Service (PHS) policy for the Humane Care and Use of Laboratory Animals. The University has an Animal Welfare Assurance on file (assurance number D16-00435) with the NIH Office of Laboratory Animal Welfare. The rabbits were housed at the animal facility, which is registered with the US Department of Agriculture and undergoes annual inspection by that agency, for a mandatory 14-day acclimatization period before the study and throughout the 42-day study period. The rabbits were given free access to water and food with 12-h cycles of light and dark under centrally controlled and monitored ambient temperatures, airflow, and pressure differentials. There were 5 rabbits assigned to the experimental group (mesh implants) and one rabbit assigned to the control group (sham-operated without mesh). All rabbits were anesthetized using acepromazine 1.1 mg/kg and isoflurane in oxygen 4–5%.

At implantation, each rabbit was operated on using a retromuscular repair, as previously described in a rabbit model [55], to mimic the totally extra-peritoneal (TEP) surgical technique commonly used in hernia repair in humans. After skin asepsis, a 5 cm incision was made in the ventral midline of the abdominal wall, and the abdominal cavity was carefully entered along the linea alba. Just lateral to the linea alba, the posterior fascia was incised to the level of the rectus muscle, and the posterior sheath was carefully peeled off of the rectus muscle laterally. At the semilunar line, the dissection transitioned to the plane between the transversus abdominis and internal oblique muscles laterally. An intermuscular plane was created to accommodate a 3 × 3 cm piece of mesh (Figure 3). One coupon of the PGMA/HSA grafted PP mesh and one coupon of the unmodified PP mesh were implanted into the intermuscular pockets on either side, symmetrical with respect to the linea alba. The PGMA/HSA grafted PP mesh coupon was secured to the muscle with 540 µL of the bifunctional poloxamine hydrogel adhesive delivered via a pipette and applied across the mesh surface. The unmodified PP mesh coupon was secured to the muscle with Tisseel^®^ fibrin sealant applied across the mesh surface with the provided applicator. The rabbit in the control group was incised as described but not implanted with mesh. The anterior fascia was then closed in the midline with 3–0 polydioxanone suture in a running fashion and the skin was sutured with 3–0 polydioxanone sutures.

The rabbits were euthanized on day 42, and the skin was resected to expose the implant sites. The implant sites were photographed in situ with a transparent calibration grid overlay to assess mesh slippage and contraction (Figure 4). The mesh and surrounding muscular tissue margins were immediately (within 15 min) harvested en bloc. Each mesh–tissue block was cut into four 1 × 3 cm sized sections for mechanical testing (n = 2 with mesh and n = 1 muscle tissue margin without mesh) and histological analysis of biocompatibility (n = 1). Sections for mechanical testing were maintained between two gauze pads soaked in PBS to maintain moisture and immediately placed on ice. Sections for histological analysis were submerged in 10% neutral buffered formalin.

#### 4.2.10. Mesh Slippage and Contraction

Mesh slippage and contraction at the implant sites were grossly assessed in situ immediately after euthanasia and skin resection and were visualized using calibrated digital images (300 DPI) acquired under ambient lighting conditions (Figure 4). Mesh slippage and contraction were defined as gross bunching in the mesh causing distortion of the usual flat profile of the mesh. Visual evidence of bunching was assessed in each of nine 1 × 1 cm grid zones denoted by the transparent grid overlay and graded using a semi-quantitative method. Mesh with distortion in 1 or fewer zones (that is, ≤11% of the mesh area was distorted) was graded as having no bunching. Mesh with distortion in 2 or more zones (that is, ≥22% of the mesh area was distorted) was graded as having notable bunching.

#### 4.2.11. Mesh Fixation

**Figure 12:**
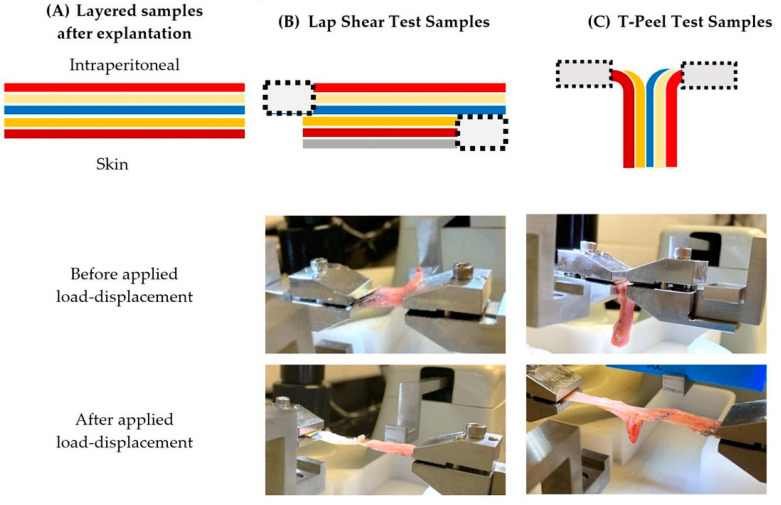
Sample mounting configurations for mechanical testing. (**A**) The sections consisted of tissue–mesh–adhesives layered composites with mesh (depicted as a blue line) and adhesives (depicted as yellow/orange lines) layered between the sub-dermal and intraperitoneal muscle tissue (depicted as red lines). (**B**) For lap shear testing, the sub-dermal muscle of each section was mounted to a 1 cm × 3 cm aluminum sample holder (depicted as a gray line) and then firmly clamped into the test rig, and the opposite edge of the mesh with intraperitoneal muscle was firmly clamped to the load cell fixture. (**C**) For T-peel testing, the sub-dermal muscle and the mesh with intraperitoneal muscle were each firmly clamped directly into the test rig and load cell fixtures. Mounting clamps (depicted as gray boxes with dashed outlines) were oriented as needed for each test.

The adhesive strength was measured consistent with ASTM F2255-05 (uniaxial lap shear testing) and ASTM F2256-05 (uniaxial T-peel testing) using a 23 N load cell (BioTester, CellScale, Waterloo, ON, Canada). Sections designated for mechanical testing were tested within two hours of euthanasia. Sections were quickly removed from the cold PBS-saturated gauze, and all testing was performed at 37 °C. For lap shear testing, the sub-dermal muscle of each section was mounted to a 1 cm × 3 cm aluminum sample holder and then firmly clamped into the test rig, and the opposite edge of the mesh with intraperitoneal muscle was firmly clamped to the load cell fixture (Figure 12). For T-peel testing, the sub-dermal muscle and the mesh with intraperitoneal muscle were each firmly clamped directly into the test rig and load cell fixtures (Figure 12). A ramp load was applied at a constant rate of displacement (10 mm/min) with continuous recording of both load and displacement.

The adhesive strength was defined as the peak load divided by the gauge length measured at the tissue/mesh/adhesive interface. The gauge length was the contact area of the mesh–tissue interface for lap shear testing and the contact width of the mesh–tissue interface for T-peel testing. The maximum load required to disrupt the mesh from the tissues was recorded, and the adhesive strength was calculated for n = 2 mesh types per rabbit and n = 1 muscle tissue margin without mesh per rabbit. The type of test performed was determined by the amount of tissue available for clamping, but some sections could not be tested (e.g., too small for mounting), so not all rabbits had all data points.

#### 4.2.12. Mesh Biocompatibility

Specimens designated for histology were cut into two 1.5 cm pieces, placed into tissue cassettes, and processed for paraffin embedding using an automated tissue processor. Microtomed sections (5 μm thick) were mounted on glass slides and stained using standard histological protocols. Stains included hematoxylin and eosin to identify signs of an inflammatory response and foreign body reaction, Masson’s trichrome to identify fibrous connective tissues and degree of collagen synthesis, and Herovici’s stain to differentiate immature and mature collagen. The host tissue response was quantitatively graded in a blinded manner.

Representative digital images of the stained slides (4 images per mesh per rabbit for each stain) were generated using a transmitted light microscope (BA410, Martin Microscope Company, Easley, SC, USA) equipped with a digital camera (Motic M14, Martin Microscope Company, Easley, SC, USA). We acquired images at 2.5× magnification that were subdivided into a grid of 64 regions and then used a random number generator to select 8 individual regions for quantitative analysis.

Selected regions were imaged at 20× magnification and analyzed using image analysis software (NIH ImageJ, Bethesda, MD, USA). Images were post-processed using preset algorithms (“background subtract” and “adjust levels” to accommodate variations in light microscopy and provide uniform brightness. Hue analysis was performed based on published methodology [55]. Briefly, independent, blinded observers used color deconvolution to separate the RGB images into three color channels. In sections stained with hematoxylin and eosin, red/pink colors were masked, and purple/black pixels representing the macrophage nuclei were counted. In sections stained with Masson’s trichrome, pink/red pixels representing muscle tissues and blue pixels representing collagen were counted. In sections stained with Herovici, red pixels representing mature collagen and blue pixels representing immature collagen were counted. Using the software, the percent area of each tissue type was calculated using the number of counted pixels. The inflammatory response and collagen synthesis were thus determined for mesh fixed with the bio-adhesive mesh system and mesh fixed with fibrin sealant in each rabbit.

## Figures and Tables

**Figure 9:**
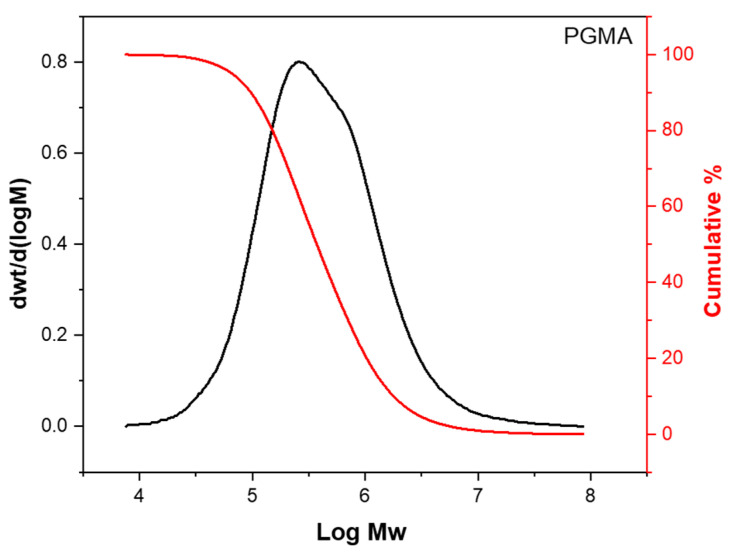
PGMA molecular mass (Mw) distribution obtained with GPC.

**Table 1:** Average adhesive strength at the tissue/mesh/adhesive interface after 42 days implantation for the fibrin-fixed PP meshes and the bio-adhesive mesh system. Tissue margins without mesh were tested as controls.

Rabbits	Test	Fibrin	Bio-Adhesive	Tissue Margins without Mesh
R1–R3	Lap Shear	6.8 ± 1.1 N/cm^2^	7.7 ± 2.9 N/cm^2^	7.3 ± 2.2 N/cm^2^
R4–R5	Peel Test	1.6 ± 0.4 N/cm	3.0 ± 1.8 N/cm	2.3 ± 1.4 N/cm
R6 (control)	Peel Test			4.5 N/cm

**Table 2:** Average percentage area of individual tissue components in histological sections (n = 4 images/mesh/rabbit for each stain).

Stain	Tissue Type	Fibrin-Fixed	Bio-Adhesive	Control
Hematoxylin & Eosin	nuclei	9.0% ± 1.8%	11.3% ± 1.0%	4.7% ± 2.5%
Masson’s Trichrome	collagen	41.3% ± 10.1%	44.3% ± 10.5%	28.3% ± 5.3%
	muscle	20.3% ± 5.3%	13.7% ± 6.6%	26.7% ± 11.7%
Herovici	immature collagen	13.4% ± 4.5%	13.4% ± 4.1%	11.9% ± 4.7%
	mature collagen	17.2% ± 3.0%	16.3% ± 3.6%	16.8% ± 4.9%

## Data Availability

All data are available upon request from the corresponding author.
